# Implementing living evidence to inform health decisions: A strategy for building capacity in health sector (Protocol)

**DOI:** 10.12688/openreseurope.14041.2

**Published:** 2022-05-20

**Authors:** María Ximena Rojas-Reyes, Gerard Urrutia Chuchí, Gabriel Rada, Pablo Alonso, David Rigau Comas, Ariadna Auladell-Rispau

**Affiliations:** 1 Institut d'Recerca-Servei d'Epidemiologia Clínica i Salut Pública, Hospital de la Santa Creu i Sant Pau, Carrer de Sant Quintí, Barcelona, 08041, Spain; 2 Biomedical Research Institute Sant Pau (IIB Sant Pau), Carrer de Sant Quintí, Barcelona, 08041, Spain; 3 Consorcio de Investigación Biomédica en Red de Epidemiología y Salud Pública (CIBERESP), Av. Monforte de Lemos, 3-5. Pabellón 11. Planta 0, Madrid, 28029, Spain; 4 Cochrane Iberoamerica, Hospital de la Santa Creu i Sant Pau C/ Sant Antoni Maria Claret, 167, Pavelló 18, planta 0, Barcelona, 08025, Spain; 5 Epistemonikos Foundation, Av. Holanda 895. Providencia, Santiago, Chile; 6 Faculty of Medicine, Pontificia Universidad Católica de Chile, Av Libertador Bernardo O'Higgins 340, Santiago, Chile

**Keywords:** living evidence, evidence synthesis, living evidence framework, health decisions, decision making, knowledge transfer, capacity building, heath systems research

## Abstract

Every day important healthcare decisions are made with incomplete or outdated information about the effects of the healthcare interventions available, what delivers the best value for the health system and where more research is needed. It is necessary to invest in strategies that allow access to reliable and updated evidence on which to base health decisions.

The objective is to develop and evaluate a strategy for building the capacity among different actors of a country’s health system to implement the model known as “Living Evidence” [LE] in the evidence synthesis and dissemination of knowledge transfer [KT] products to inform health decisions. The study will involve professional members of health system organizations in charge of developing KT products to inform health decisions.

The project will be developed in three complementary phases: 1) LE-implementation framework development through review of the literature, brainstorming meetings, user testing, and expert consultation; 2) training in LE tools and strategies; 3) developing LE synthesis for KT products by applying the framework to real-life diverse situations.

To achieve the capacity-building strategy assessment goal, several surveys and interviews will take place during the process to assess: 1) the LE-implementation framework for the incorporation of LE synthesis in the development of KT products; 2) the training workshops; 3) the whole capacity-building strategy used for health system organizations be able of implementing the LE as part of the KT products they regularly produce.

The expected results are an effective capacity-building strategy for health system organizations to implement the living evidence model in different KT products; a LE-implementation framework to be applicable to any country or region to incorporate LE in the KT products; LE synthesis for KT products directly applicable to the real-setting situations; integration of Epistemonikos-L.OVE platform for keeping the LE process in the development and updating of KT products.

## Plain language summary

“ *Living Evidence to inform health decisions*” is a research project involving the design and evaluation of a model strategy to generate, use, and apply innovative methods and tools to support health decisions to be based on the most recent evidence.

“Living evidence” (LE) refers to the methodological approach that permits new research findings to be continually incorporated to evidence synthesis as they become available. LE is optimal to ensure a rapid update of products that inform on the effects of controversial health interventions, and/or clinical practice guidelines’ recommendations, where there are uncertainties.

This project seeks to build capacity among health sector organizations working to support health decision making, in the incorporating of the LE approach to the resolution of relevant and rapidly changing clinical questions. We will identify health technology assessment agencies, guideline-developing organizations and secondary and tertiary health care institutions (hospitals) from Spain and Europe. The target population is professionals, members of the technical teams of these organizations, in charge of developing evidence syntheses products aimed at informing clinical and/or policy decisions. To achieve our goal, we will conduct three complementary phases aimed at, developing a framework that will help organizations in the process of implementing the LE approach in the products they usually develop to support health decision-making; training members of the participant organizations in LE methods, tools and strategies; and, reinforcing knowledge and capacity for producing and using LE synthesis among participants, by applying the acquired knowledge, tools and the framework in various real-life situations.

Surveys and interviews will take place during the process to assess the LE-implementation framework, the training workshops and the whole strategy used for building capacity.

The expected results include an effective capacity-building strategy for health system organizations to implement the living evidence model in their current process and a LE-implementation framework to guide them in this task.

## Introduction

Every day in the world, important healthcare decisions are made with incomplete or outdated information about the effects (benefits and harms) of the different healthcare interventions available, what delivers the best value for the health system, and where more research is needed. Evaluating the best available evidence as a whole on a given health problem to make well-informed clinical and health policy decisions is increasingly challenging as, in many cases, published research is abundant but also of poor quality ^1, 2^, and despite increasingly stringent regulations, fraudulent or biased information remains. Therefore, basing clinical and health policy decisions on reliable evidence that is easily accessible and updated requires sophisticated and laborious processes of identification, critical evaluation, and synthesis ^3^. It is also necessary to have a mechanism (or system) that ensures the process meets desirable characteristics such as rigor, systematization, and reproducibility in order to obtain valid conclusions that can guide health decision-making.

The current economic situation imposes the need to increase efforts for achieving greater efficiency in allocating healthcare resources that guarantee the sustainability of the health systems. This situation merits the countries to invest in strategies that easily allow them to have reliable and updated evidence on which to base health decisions (e.g. clinical decisions, decisions for public health and coverage), increasing the value of care and of available resources.

Currently, there are newly developed and validated innovative technological tools for systematic identification, selection, and comprehensive storage of evidence, that facilitate producing overviews of the available evidence on a given topic more efficiently than the current process, with the advantage of being constantly updated ^4, 5^. These tools have the potential of allowing health professionals to base their decisions on the most recent evidence. However, despite constant advances in the appropriation of scientific knowledge and technological developments, there is still a gap among healthcare professionals in producing and using the most current evidence for decision-making ^6, 7^. There is a need to strengthen the capacity of the different actors of the health system to better leverage the methodological developments and existing technologies in a fast and efficient manner on which to base their decision-making process.

With this in mind, we have assembled a research proposal that aims to develop and evaluate a strategy for building the capacity among different actors of a country’s health system, such as physicians, researchers, healthcare professionals in training, members of health technology assessment agencies, and guideline developers, to implement the strategy known as “Living Evidence” (LE) ^8^, in the evidence synthesis and dissemination of knowledge transfer products [KT products] they usually work on (e.g. structured evidence summaries for policies [SES], health technology assessment reports [HTA], and recommendations in Clinical Practice Guidelines [CPG]) for decisions to be based on must current evidence.

The project is based on previous and recent developments achieved by several methodological research groups and networks, such as the LE model ^8– 12^, the GRADE approach ^13^, rapid overviews ^14^, SUPPORT evidence summaries ^15^, and the Epistemonikos project ^16^ and its technological tools ^17^.

### Live Evidence model

Over the last six years, members of the Cochrane Collaboration and a number of its international partners have developed the foundations of the Living Evidence model for evidence synthesis and dissemination of systematic reviews [SRs]. Living reviews are SRs that are continually updated as new evidence appears ^9^. The production of living reviews begins once the SR has been developed under its traditionally known quality standards (baseline review), which guarantee that the methodological approach has been the most appropriate and has ensured control of biases. Currently, the methodological approach, as well as the model for the production of living SRs are described in a series of articles ^9– 12^ and have been tested and adopted by some review groups within and outside Cochrane.

### The GRADE approach

SRs and overviews of the effects of healthcare provide essential but not sufficient information for making well informed decisions. Reviewers and those who use reviews draw conclusions about the quality of the evidence, either implicitly or explicitly. Such judgments guide subsequent decisions. The GRADE approach ^13^ is a systematic, explicit and transparent approach to making judgments such as these that can help to prevent errors, facilitate critical appraisal of these judgments, and improve communication of this information. The evaluation of the certainty in the evidence with GRADE is now days part of any structured report in which evidence is used to support healthcare decisions ^18, 19^.

### The "overview"(panoramic reviews of the same topic)

Nowadays, it is not uncommon to find more than one published SR that answers the same question, often reaching different conclusions ^20, 21^. On the other hand, the continuous development of health technologies has led to more than one intervention competing for the same health problem ^22^. Traditional SRs that are focused on a particular intervention or a limited range of them provide a partial picture of the knowledge necessary to identify the best option available. It is common to find more than one SR related to the same health problem, evaluating different interventions. The overviews (also known as "review of reviews" or “scoping reviews”) seek to evaluate the effectiveness of all the interventions available (for prevention or treatment) for a given health condition in order to make an integrative comparative synthesis of the evidence and draw conclusions about which is more effective and safe for the patient ^14^. They are based on the SRs that have been developed to assess each particular intervention of interest. Frequently, as part of the overview development, it is necessary to update the meta-analyses of the original SRs when new eligible studies are found. Sometimes, overviews lead to network meta-analyses since reviews that share the same comparator (i.e. placebo) are frequently identified for different interventions with no studies comparing them directly with each other ^23^. The final results of the overview will allow for assessment of the quality of the existing body of evidence and to draw conclusions about the effectiveness and safety of all interventions addressed.

### Structured evidence summaries

For several years, there has been considerable interest in structured evidence summaries to inform decision makers, especially for public policy. One of the most recognized efforts in this regard was the SUPPORT project ^24^. From this project emerged structured formats to briefly inform the key aspects of the available evidence on a defined topic or PICO question (i.e. a structured way to define clinical questions with a clear definition of population, intervention, comparator and outcomes of interest). The SUPPORT summaries contain a brief overview of the problem, a list of key messages presented in short sentences, and the summary of evidence found for safety and effectiveness outcomes with the results of the evaluation of its quality based on GRADE ^25^.

### Epistemonikos project and the L.OVE platform tools

The Epistemonikos Evidence Synthesis Project [Epistemonikos-ESP] is a collaborative initiative established in 2012 with the objective of collecting, organizing and comparing all relevant research evidence for health-related decision-making, through a friendly and multilingual interface ^16^. Currently, its database includes more than 100,000 SRs and hundreds of thousands of individual studies. In addition to identifying almost all of the existing SRs, the Epistemonikos database allows the comparison of different SRs for the same question by displaying an Evidence Matrix, a dynamic table that shows the SRs and all the studies included in these reviews. This matrix is constantly updated as part of the Epistemonikos EPS procedures, where any new SR or single study that is published in the topic is automatically incorporated into the matrix.

Epistemonikos has also developed the platform L·OVE (Living Overview of the Evidence), which gathers all the scientific evidence relevant for a specific health topic (from primary prevention, to secondary prevention, crossing though to diagnosis and therapy), organizes it in PICO format, and keeps it up to date ^17^. Given its technical design, this platform helps researchers to identify gaps in knowledge and give priority to certain research areas. For decision makers, it offers a transverse vision on different options available and allows them to identify limitations in evidence that define decisions, all while being very user-friendly.

The above-mentioned developments will be the basis for this project thanks to a cooperative institutional effort that allows for the construction of a living evidence implementation strategy, aimed at better decision making for a country’s health, with the aim that it will be reproducible and applied to any country, region, or health system. It is expected that this will increase the impact of health research, reducing the costs and time consumption related to KT products updating processes.

## Objectives

As a research project, our main objective is to develop a capacity-building strategy to obtain, improve, and retain skills and knowledge needed to develop and use “Living evidence synthesis” among members of health system organizations in charge of developing KT products to inform health decisions.

Specific objectives are:

- To design and evaluate a framework for the incorporation of living evidence synthesis in the development of three types of KT products to inform health decisions: recommendations in clinical practice guidelines [CPG], health technology assessment reports [HTA], and structured evidence summaries [SES] for institutional or public policies.

- To build capacity among members of health system organizations in the use of innovative and effective tools to support the generation and maintenance of living evidence synthesis.

- To develop living evidence synthesis for KT products to inform health decisions in a real setting

- To assess the effectiveness and usability of the Epistemonikos-L.OVE platform as a tool for keeping the living evidence process, as part of the development and updating of the different KT products

## Methods

### Study population

The study population will be healthcare professionals and technical team members of different health system organizations in charge of developing KT products to inform health decisions. This population will be enroll for the project phases 2 and 3 (see desing). 

### Sampling

We will seek to involve three types of organizations from the health sector, that fulfill the characteristic of synthesizing evidence to develop KT products aimed at supporting health decision-making. 1) Health technology assessment [HTA] agencies; 2) secondary and tertiary healthcare institutions (hospitals) involved in institutional HTA programs and 3) clinical practice guideline [CPG] development organizations.

The identification of these organizations will be done through the networking relationships that the IIB Sant Pau has, where the Ibero-American Cochrane Center operates, which is recognized in Europe for its different developments in the area of evidence synthesis, and support for the evidence-based medicine. Its relations of interest for this project include: scientific societies and organizations of the Spanish Biomedical Research Network Consortiums (CIBER) ^26^, clinical guideline development organizations in Europe, and hospitals participating in a program that promotes evidence-based informed policies in healthcare institutions in Spain ^27^

The director or leader in charge of the evidence synthesis team from these organizations will be approached to present the project and invite them to take part. We will seek to enroll at least two organizations of each type described above, by a convenient sample.

Each enrolled organization will be asked to involve at least three members of their organization's technical team in charge of developing evidence synthesis to support decision-making. To take part in the study, the members of organizations should meet the following selection criteria:

- To be a contracted worker in the organization

- To be involved in evidence syntheses development tasks

- To complete and provide the informed consent for individual participation in the study

### Design

Research has not indicated any single design or set of approaches which is guaranteed to succeed in building capacity and improving performance ^25^. However, this project involves the elements that underpin the approach to capacity-building in health research, such as the external context considerations, the stakeholder’s involvement, the consideration of institutional/organization rules, the capability and resources, the performance and adaptation ^28^.

**Figure 1.**
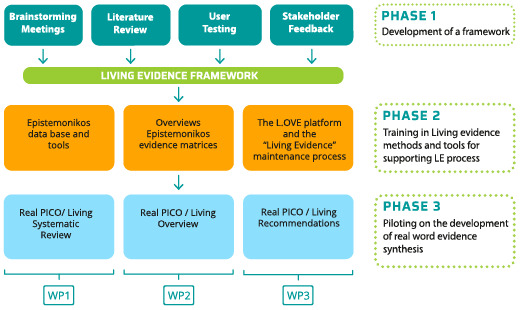
Project development overview.

The project will be developed in three independent but complementary phases (see Figure 1):

Phase 1. **LE-implementation framework development** through review of the literature, brainstorming meetings, user testing and expert consultation.

Phase 2. **Training in Living Evidence tools and strategies** through the participation of members from different organizations in online workshops.

Phase 3. **Developing living evidence synthesis for KT products** by applying the framework as well as the knowledge obtained in training workshops to real-life diverse situations.

Information necessary to assess the capacity-building strategy, the LE implementation framework, and the use of the Epistemonikos tools for keeping the evidence “living”, will be collected throughout phases 2 and 3. The results of these assessments will allow the strategy and the LE-implementation framework to be redefined and improved as a tool to incorporate and maintain living evidence in the KT products that the participating organizations regularly produce. 

The project has organized its empirical work around three work packages (WPs), each aimed at a different group or type of KT products they develop:

*WP1. Living evidence syntheses.* This seeks primarily to build the capacity among professionals from scientific organizations, hospital institutions, and HTA agencies to produce living evidence synthesis to inform healthcare decisions.

*WP2. Structured summaries of evidence*. This seeks to build the capacity among professionals from hospital institutions and HTA agencies to prepare structured evidence summaries based on living overviews using the Epistemonikos tools, to inform decision-makers.

*WP3. Living guideline recommendations.* This aims to build the capacity among developers of CPG to produce living evidence synthesis to inform key recommendations within a guideline and update guidelines through the development of living overviews supporting by the Epistemonikos tools.

### Phase 1: Framework development

An initial framework (LE-implementation framework) will be developed aimed to guide groups to use appropriate criteria for defining which clinical problems (structured into clinical questions) benefit from a constant review of the new evidence, the frequency with which these processes should be carried out, and whether to incorporate the new evidence synthesis and conclusions in the KT products this evidence supports (i.e. CPG, HTA, and structured evidence summaries for health policies). The framework will be designed as a tool for organizations to apply in the usual processes they follow to develop evidence synthesis. A set of instructions will be provided for each framework section. Nevertheless, we assume the potential user has basic training in evidence synthesis methodology, as the minimum requirement that professionals who work in this area are expected to have.

For this purpose, four complementary actions will be carried out: 1) review of the methodological articles generated on the subject; 2) brainstorming meetings; 3) a user testing among potential users; and 4) consultation with expert methodologists from internationally recognized groups working in the field of living evidence synthesis for evidence-based recommendations.

It is expected that the final framework can be suitable to be used by organizations from any country or region; therefore in its development as well as its evaluation, we will involve international expert advisors, health sector organizations from different countries in Europe and potential users from around the world.

***1. Review of methodological articles***

We will perform a survey based in a literature review to identify methodological articles about living evidence synthesis. A methodological article is one that presents new approaches, changes to existing methods or the discussion of quantitative and data analytic approaches to the research community ( Mvorganizing). To be considered a “methodological article” for this review, it should include: an overview of methods; the main elements of the proposed methods; the breadth of application for the proposed methods for statistical procedures; and a summary of some of the essential feather. No other selection criteria will be applied.

We will search for methodological articles in MedLine (via PubMed) and Google scholar using the following free text words: “ *Living evidence*”, “ *Living systematic review*”, “ *Living systematic reviews*”, “ *Living metanalysis*” and “ *Living evidence methodology*”. The information specialist of the Iberoamerican Cochrane Center, will devise the search strategy. The search results will be screened by two independent reviewers that will classify them into two groups based on titles and abstracts: a) methodological articles presenting the living evidence approach or b) articles reporting living evidence synthesis (e.g., LSR, living network-metanalysis (LNMA)). For the purpose of this particular review only the methodological articles will be selected. The two reviewers will extract data on main elements of the proposed methods and technical considerations presented by the authors and collected in a previously designed data extraction sheet, available in the project repository. Data obtained from these papers will be grouped into categories related to the type of information and the moment in the evidence synthesis process in which this information can be applied. We will perform a descriptive analysis of the data extracted and presented the results in a combination of summary tables and narrative descriptions. Data obtained from these papers will be grouped into categories related to the type of information and the moment in the process in which this information can be applied. This review results will contribute to the development of a preliminary checklist of key methodological aspects that will be used as the starting point for the development of the living evidence implementation framework for health system organizations to use and incorporate the LE methodology.

***2. Brainstorming meetings***

We will organize two brainstorming meetings to generate ideas and solutions to the identified challenges on developing living evidence synthesis. Participants of these meetings will be the lead research group from the Biomedical Research Institute Sant Pau (IIB Sant Pau), Iberoamerican Cochrane Center and members from the Epistemonikos group. Both groups have professional experience and previous knowledge on evidence synthesis, developing knowledge transfer products and frameworks for health decision making.

In advance to the meetings, participants will receive the results from the previously described methodological review. In the first meeting we will collect the ideas given by the participants on the following aspects: i) structure of an implementation framework, ii) methodological elements to be included in the framework, and iii) guidance questions to be included.

Based on the suggestions obtained during this meeting, we will develop the first draft of the framework. We will structure the framework in sections according to the time in which they must be applied during the evidence synthesis process (i.e. at the time of defining the relevant questions to be answered through a living evidence synthesis; when planning the evidence synthesis; during the evidence surveillance and monitoring process, when integrating new eligible evidence and at the time of publication of updates, etc.). Each section will include a series of guidance questions defining each step of the process. In a second meeting, we will present the draft of the framework in order to collect new ideas and innovative suggestions for its improvement. 

***3. User testing***

We will invite up to 10 participants from the Cochrane Collaboration, members of evidence synthesis related networks and members of potential participant organizations or other potential users to take part in the user testing. This step seeks to evaluate the comprehension of: framework’s structure; the guidance notes and instructions; the relevance of the guiding questions of each section; clarity of questions, statements and instructions, among other issues. We will ask participants to take one of the questions they are working on for developing evidence synthesis and apply the framework for planning this evidence synthesis. A structured evaluation form will be provided for users to register their evaluations and comments. Results will be collected and summarized in an Excel (version 16.34) database where notes and comments will be also transcribed. Final results will be reviewed in two or more research team meetings in order to decide whether or not to incorporate changes into the framework. A new version of the LE-implementation Framework will be generated and undergo experts’ revision.

***5. Consultation with expert methodologists***

As part of the planning phase, a group of international expert advisors will be made-up from members of the Cochrane Living Systematic Review Network, Australian Living Evidence Consortium, GRADE working group, the Guideline international network (G-I-N), the National Institute of Clinical Excellence (NICE), and Mc Master Health Systems Research Forum, among others working in the field. This group will be in charge of reviewing the LE-implementation Framework generated from the previous steps. Their comments and contributions will be obtained by individual interviews and e-mail rounds and will be integrated into the framework to generate a final version to be applied by participating organizations in the subsequent phases of the project.

### Phase 2: Training in Living Evidence tools and strategies

This phase will run concurrently with phase1, once we have completed the recruitment process of participating organizations and their members

For this project, the Epistemonikos L.OVE platform will be the tool used as part of the strategy to keep the living evidence (i.e., for generating and maintaining the living evidence process). L.OVE is a digital tool that combines a series of technological advances (including artificial intelligence algorithms) with the effort of a network of experts, to obtain and organize health evidence as soon as it is produced. A L.OVE is created for each health topic or condition (e.g., chronic obstructive pulmonary disease) and the health questions are organized by specific subtopics, such as prevention, diagnosis, therapy, or prognosis creating a comprehensive map of questions based on the PICO format (population, intervention, comparisons, and outcomes).

This platform has been chosen as the technological enabler in this project because it has several potential advantages for supporting a living evidence process that will be tested as part of the project, such as: even though it gathers information from 10 sources that are routinely examined in the Epistemonikos Database it can be programmed to search other databases relevant to the specific topic; once the PICO question is included in the L.OVE platform, the searching results are obtained very quickly (between one minute to a couple of hours); the information from saved questions is constantly updated as new evidence appears; users can create alerts when new evidence appears; the screening and selection of evidence processes can be shorter than usual thanks to artificial intelligence. The Epistemonikos -L.OVE platform has been extensively used and its effectiveness in supporting Living Evidence processes has been validated during the COVID-19 pandemic ^29, 30^

Therefore, this phase seeks two main objectives: 1) to train participants in the Epistemonikos tools and L.OVE platform and, 2) to evaluate the strategies used for this training.

A set of training workshops will be carried out, aimed at the members enrolled from each participating organization. Training will be focused on the processes inherent to generating living evidence based on the Epistemonikos L.OVE platform. Complimentary workshops for supporting the evaluation of certainty of updated evidence, according to the GRADE approach, will be offered depending on the degree of experience and previous training of the participants.

### Phase 3. Developing real word living evidence synthesis for KT products

This phase seeks to apply the LE-implementation framework as well as the knowledge obtained in training workshops to real-life diverse situations. According to the particular interest of the participating organizations, they can work on any of the following KT products: i) structured evidence summaries for institutional and/or public health policies; ii) health technology assessment reports, and iii) evidence-based recommendations for a CPG.

**Figure 2.**
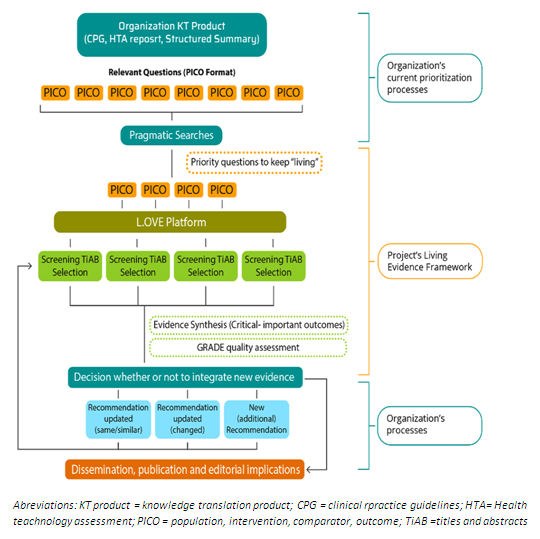
Workflow for developing living evidence synthesis for priority questions of a given KT product.

**Figure 3.**
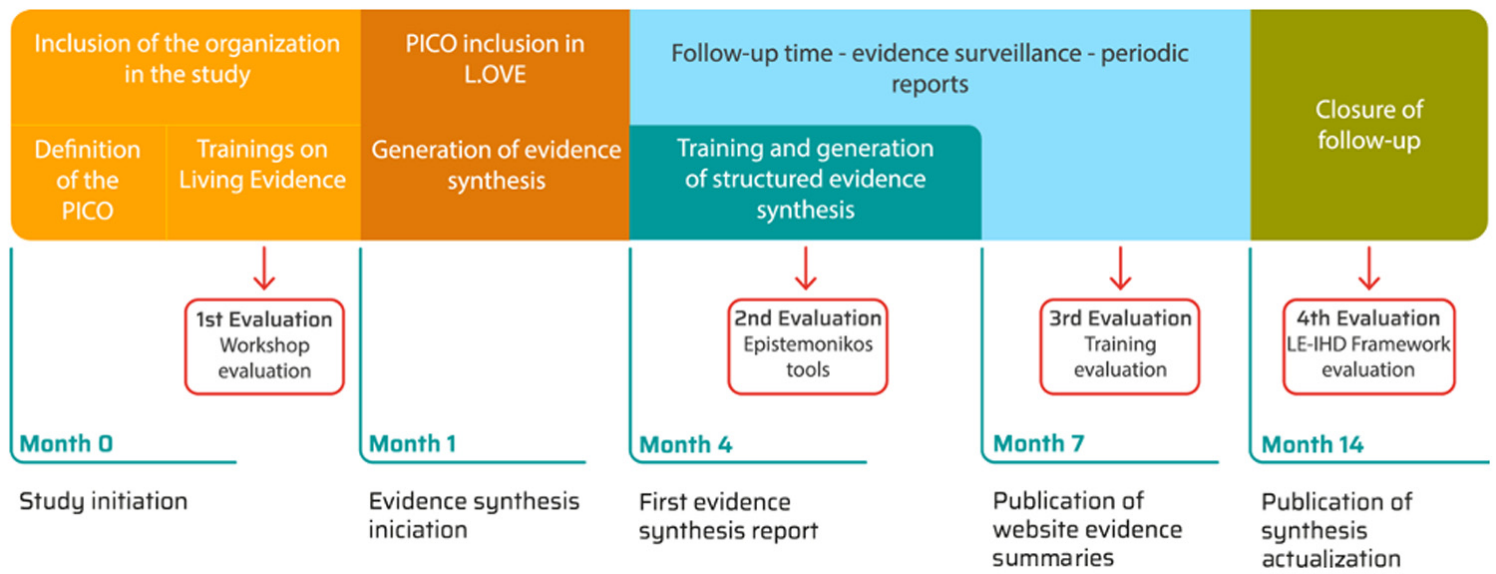
Timeframe for study follow-up and data collection (key evaluations).

Following the principle of “learning by doing” ^31^, we expect members from the participating organizations to generate at least one living evidence synthesis (i.e., one PICO) needed to develop their own KT products following the LE-implementation framework (see Figure 2 and Figure 3). To support this task, and ensure that all the organizations' teams are receiving the same information and detailed instructions, a Manual of standardized operating procedures will make available to the participants in an online repository or as a virtual document. In this way, the participant’s skill development will be strengthened through the experience while we evaluate the LE-implementation framework performance.

Each evidence synthesis will be worked on as an independent project, with an assigned working group that will involve content experts (physicians) and methodological experts from the participating organization (i.e., HTA agencies, guideline development groups, scientific organizations, research consortiums, hospital institutions) and a member of the research team (i.e., IIB Sant Pau and Epistemonikos Foundation).

### Data collection and analysis

Several measures will take place during the whole project in order to assess: the LE-implementation framework; the training workshops on Epistemonikos tools; and the capacity-building strategy used for participant health system organizations be able to implement the “living evidence” process in the development of KT products. All the participants will be invited to answer the questionnaires or the in-depth interviews to complete these assessments. The evaluations will be limited to what is necessary to meet the study objectives. Figure 3 presents the timeframe and when evaluations will take place.

The expected size of the data collected for this assessment will be given by the number of participants who join the project from each organization.

***Evaluation 1. Assessment of training workshops***

After finishing each training session (workshop) participants will be asked to answer anonymous online surveys to assess the workshop’s materials (i.e. learning guide and objectives, support instructions and tutorials), training activities as well as the skills of the teacher who led the session and the presentation that he/she used to present the key concepts and procedures. The structured questionnaire will ask the participants to indicate his/her level of agreement (from strongly agree to strongly disagree), using a five-point Likert scale, with a series of statements that will allow us to evaluate the relevance and appropriateness of the aspects of the training workshop.

Data obtained from these surveys will be analyzed using summary measures (e.g. proportion of participants who answer each of the five Likert scale options). These data will allow researchers to redefine this part of the capacity-building strategy (if necessary). Training workshops can be also re-evaluated accordingly.

***Evaluation 2. Effectiveness and usability of the Epistemonikos tools and L.OVE platform***

All members of technical teams and task forces taking part in the study will be invited to respond to an online survey once the initial evidence synthesis process has been completed. The survey will be anonymous but will collect information about:

- Individuals' demographic characteristics and background (e.g. age, academic degree, amount of experience)

- Previous experience in developing evidence synthesis and/or systematic reviews

- Knowledge and previous experience planning and performing literature searches

- Knowledge and previous experience completing the literature screening process

This survey will evaluate the usability and performance of the Epistemonikos L.OVE platform as a tool for evidence identification, screening and maintenance of the living evidence process. The information asked to assess performance will be limited to that directly related with the tasks participants must complete by using the Epistemonikos L.OVE tools, such as planning of evidence synthesis and evidence monitoring and surveillance (including screening, classification and study selection). The information asked to assess usability will be based and limited to participants experience using the tools for the tasks mentioned above. The survey will ask participants to indicate his/her level of agreement (from strongly agree to strongly disagree), using a five-point Likert scale, with a series of statements that will allow us to evaluate the usability and performance of the tools.

Data obtained from this survey will be analyzed using summary measures (e.g. proportion of participants who answer each of the five Likert scale options).

***Evaluation 3. Assessment of the capacity-building strategy***

To achieve this objective, two complementary approaches will be used for collecting data: an online survey to all the participants and in-depth interviews to a randomly selected number of participants. The online survey will be conducted at six months after the evidence surveillance period has started. All members involved in the evidence synthesis development from participant organizations will be invited to respond to the online survey. Data to be collected in this survey will be limited to that necessary for evaluating:

- Clarity, relevance and timeliness of weekly messages sent to participants informing tasks and procedures.
- Clarity, completeness and utility of the Manual of Standardized Operating Procedures and its instructions.
- Clarity and timeliness of the support given by the expert researcher assigned to the group.
- Clarity and timeliness of solutions provided to doubts and difficulties that arises during the process.
- Satisfaction with results obtained (as individual and as member of the organization group) by developing the living evidence synthesis under the learning by doing methodology.

At the end of follow-up (i.e. the end of the PICO question evidence monitoring) we will perform semi structured in-depth interviews to a randomly selected sample of participants. The randomization will be performed as a stratified random sample (including two groups/strata: a) technical team and/or taskforce members and b) team leaders), to guarantee that members from each group will be represented in the sample. This will be performed using Excel (version 16.34) software. These interviews seek to obtain information about participants´ perception of:

- The capacity-building strategy (i.e., training workshops, instructions and tutorials, accompaniment in “learning by doing” process, among other activities completed to refine the strategy within the study progress).
- The LE-implementation framework and its effectiveness to guide the incorporation of living evidence synthesis processes into their usual working tasks (either for producing KT products or other evidence synthesis process to inform decision makers).

The interviews will be carried out by a third-party expert in this type of interview, with a script of questions generated by the research group. The data obtained from these interviews will be included anonymously in the Nvivo® (version 12) ^32^ program by the same third party and analyzed as qualitative data by an expert in the subject.

***Evaluation 4. Assessment of the LE-implementation Framework***

As presented before, the in-depth interviews will serve to evaluate the LE-implementation framework and its effectiveness in guiding the incorporation of living evidence synthesis processes into the participant’s usual working tasks in a sample of participants. To obtain information from all the participants, an online survey using Google Docs will be conducted prior to these interviews. Two types of questionnaires will be developed for this end, one for organizations technical team members and the other for organization’s group leader.

*-Technical teams' questionnaire:* This questionnaire will have a set of questions assessing participants experience with the use of the LE-implementation framework for planning and guide the living evidence synthesis.

*- Group leaders’ questionnaire:* This questionnaire will address the comprehension of the framework and its “usability” as a tool for incorporating living evidence synthesis in the current process of the organization. We will include a set of questions that permit the evaluation of how developers are taking into account the information and guidelines presented in the LE-implementation framework and/or following the pathway for guiding the inclusion of the new evidence in the already existing KT products.

Both questionnaires will be structured, asking participants to select answer options and/or to indicate his/her level of agreement (from strongly agree to strongly disagree), using a five-point Likert scale, with a series of statements that will allow us to evaluate the above-described aspects of the framework and capacity-building strategy.

Data obtained from these surveys will be analyzed descriptively using summary measures (e.g. proportion of participants answering each item option in categorical variables and median or mean with interquartile range or standard deviation for continues variables).

### Expected results

This project will generate different types of results:

- An effective capacity-building strategy to implement the living evidence model in different KT products.

- The generation of living evidence synthesis for KT products directly applicable to the real-setting situations.

- A LE-implementation framework to incorporate living evidence in the KT products that can be applicable to any country or region.

- The integration of the Epistemonikos-L.OVE platform as a tool for keeping the living evidence process, as part of the development and updating of GPC, ETS, SES to inform health decisions.

## Conclusion

This protocol seeks to design and assess a capacity-building strategy for different organizations of a healthcare system in charge of developing KT products, to be able to use LE synthesis as part of their daily work to inform key health decisions on topics in which the evidence is rapidly evolving.

As part of the capacity-building strategy we are going to develop a framework which seeks to guide the developers of evidence synthesis for clinical practice guidelines, health technology assessment and structured evidence summaries for policies in the step-by-step process of incorporating the LE model. The framework will be based on previous developments that will be incorporated through the following actions: 1) review of the methodological articles generated on the subject; 2) brainstorming meetings; 3) a user-testing among potential users from around the word; and 4) consultation with expert methodologists from internationally recognized groups working in the field. This process will allow us to obtain a framework that may be used by organizations from any country or region ,given the participation of international stakholders (advisors and potential users) in its development.

Furthermore, we are going to test the Epistemonikos tools and its L.OVE platform as technological enablers supporting the LE processes.

### Study status

Currently we have enrolled the organizations, and their participants have completed the training modules in LE synthesis and Epistemonikos L.OVE platform tools. Moreover, we have developed the first draft of the LE Implementation Framework that is ready to undergo the expert’s assessment for feedback.

### Dissemination

The dissemination of this project’s results seeks to achieve four objectives: 1) make the successful strategies used for building capacity in the production of LE synthesis as part of KT products, known among the community of methodologists working on synthesis of evidence to inform health decisions; 2) make the final results of the “living evidence synthesis” known to physicians and clinical experts in each area of interest; 3) promote the use of structured summaries of evidence among physicians and policy makers (from healthcare institutions and public sector); 4) promote the use of technological enablers such as the L.OVE platform and Epistemonikos tools for supporting evidence synthesis tasks among the HTA and CPG developers.

***Specific actions.*** The results related to the capacity-building strategy will be presented in the KT thematic sessions of the Cochrane Colloquium and G-I-N Conference. To reach a broader community we will contact organizers of KT events of specific interest (i.e. the Oxford Health Policy Forum; the Evidence Live Oxford and the Canadian Health System Forum) with the aim of getting a slot for presenting our results.

A paper presenting the capacity-building strategy “Living Evidence to inform health decisions” and its assessment results will be published in an international journal.

The final overviews (evidence synthesis) and evidence summaries produced by participant organizations will be published on the organizations’ websites.

A special meeting with the HTA agencies’ staff will be planned to present the results as well as protocols and procedures for developing living overviews, using Epistemonikos tools and platforms to inform decision-makers. To reach a broader public, the project results will be presented in the annual HTAi meeting.

The updated CPGs will be published for use by the scientific community according to the policy of each organization.

### Ethical issues

This project proposal has been evaluated by the Ethical Committee of Hospital de la Santa Creu i San Pau and approved by the European Commission for research actions.

The project will enroll public and private organizations that work to assist decision-making in the Spanish health system, although organizations in charge of developing clinical practice guidelines for other European countries may be included.

Participation in this project will be on voluntary basis. Healthcare professionals that belong to participant organizations will be invited to participate of the project and are free to accept or decline. No coercion to participate will be tolerated from the entities, organizations, or institutions to which potential participants belong. To ensure honesty and transparency towards research participants, a consent form for participation will be generated as part of this project and presented to the corresponding Institutional Ethics Committee for approval. The form must be signed by all those that wish to participate in the project, and they will have the option to voluntarily retire from the project at any time by contacting the principal investigator according to the information provided in the informed consent form. Both the participating organizations and their members will directly benefit from the results of this investigation.

We have verified the Epistemonikos database, legal constitution, ethical principles and regulations to ensure it respects authorship and the international regulations for data bases. Epistemonikos.org is a collaborative non-profit project maintained by the Epistemonikos Foundation. All of its content and features are available for free. Epistemonikos was constituted under international ethics standards for data bases of its kind. Content owned by Epistemonikos that is made available to users can be freely used, subject to the terms and conditions of its licensing scheme. Linked content from third parties can be used in accordance with the licensing requirements of the rightful owner of the linked content.

Participants of each working group will be the authors of the products they produce. Compliance with the guidelines set by the Vancouver Protocol on authorship that states “all authors of a conjointly authored work must certify their authorship in in accordance with the discipline’s standards and practices” will be guaranteed. To avoid that any conflict of interest affects the validity of the products, we will ask all participants to declare potential conflicts of interests before making up the working groups. For this purpose, we will use the format generated by the WHO for participants in the development of CPGs, that is international recognized and complies with the aspects of interest in the case of this project.

Potential of accidental findings: During the running of the project, it is possible that researchers may identify among research participants, gaps in the knowledge or misinterpretations of key methodological concepts that should be part of skilled workers of this type of organization. We will take actions (such as anonymizing the results received) to ensure that these accidental findings will not affect the reputation of the participant nor her/his working relations within or outside the organization.

## Data availability

### Underlying data

No data are associated with this article.

### Extended data

Open Science Framework: LIVING EVIDENCE TO INFORM HEALTH DECISIONS. https://doi.org/10.17605/OSF.IO/ZC7YX ^33^

This project contains the following extended data:

- [Evaluation of Training Modules (Questionnaire)]: surveys designed to assess the training workshops
- [Execution Plan]: presents the phases of the project, the timeframe for the participating organizations, as well as the evidence synthesis development process
- [Management Structure]: presents the coordination of the project
- [Methodological review – data extraction sheet]: to use for the review of methodological articles, to extract data of the main elements of the proposed methods and technical considerations presented by the authors
- [PRISMA-P Systematic Review]: filled out checklist for the systematic review of LE reviews.
- [Project Operations Manual (POM)]: complete manual of the project
- [Training session’s guides]: this folder contains the guides and objectives for all the training workshops.
  ▪ [Module 1. Guide and learning objectives]
  ▪ [Module 2. Guide and learning objectives]
  ▪ [Module 3. Guide and learning objectives]
  ▪ [Module 4. Guide and learning objectives]
  ▪ [Module 5. Guide and learning objectives]

Data are available under the terms of the Creative Commons Zero "No rights reserved" data waiver (CC0 1.0 Public domain dedication).

### Reporting guidelines

Open Science Framework: PRISMA-P checklist for ‘Implementing living evidence to inform health decisions: a study protocol for a strategy for building capacity in health sector’. https://osf.io/6ktjx/

Data are available under the terms of the Creative Commons Zero "No rights reserved" data waiver (CC0 1.0 Public domain dedication).
