# Supplementary material for: Implementing living evidence to inform health decisions: A strategy for building capacity in health sector (Protocol)
Source: Open Res Eur. 2022 May 20;1:114. Originally published 2021 Sep 28. [Version 2] doi: 10.12688/openreseurope.14041.2 (PMC10445929; doi:10.12688/openreseurope.14041.2)
Supplement: Peer review history

# Peer review: Reviewer response for version 2

**DOI:** 10.21956/openreseurope.16015.r29337

**Published:** 2022-07-12

**Authors:** Ingrid Kremer

**Affiliations:** 1 Maastricht University, Maastricht, The Netherlands

I have reviewed the revisions of the manuscript (protocol) titled “Implementing living evidence to inform health decisions: a strategy for building capacity in health sector”, made by the authors. The revisions and answers provided by the authors address my questions and comments made on the first version. I have no further comments.

Is the study design appropriate for the research question?

Yes

Is the rationale for, and objectives of, the study clearly described?

Yes

Are sufficient details of the methods provided to allow replication by others?

Partly

Are the datasets clearly presented in a useable and accessible format?

Not applicable

Reviewer Expertise:

Health economic evaluation studies and a general expertise in quantitative and qualitative research methodologies and systematic literature reviews.

I confirm that I have read this submission and believe that I have an appropriate level of expertise to confirm that it is of an acceptable scientific standard.

# Peer review: Reviewer response for version 2

**DOI:** 10.21956/openreseurope.16015.r29336

**Published:** 2022-06-06

**Authors:** Juan Manual Lozano

**Affiliations:** 1 Department of Medical and Population Health Sciences Research, Herbert Wertheim College of Medicine, Florida International University, Miami, FL, USA

I have reviewed the second version of the manuscript (protocol) entitled “Implementing living evidence to inform health decisions: a strategy for building capacity in health sector”, by Rojas-Reyes, *et al.*, which has incorporated modifications following the review of the first version, as well as the answers provided to my comments on the first draft. I consider that my comments have been properly addressed.

 On the other hand, I have another (minor) comment, as well as suggestions for a couple of typos.

1. **Description of working packages / WP3 (page 6 of pdf).** This package is titled “Living guideline recommendations”. However, according to the description the aim is to “produce living evidence synthesis to inform key recommendations within a guideline and update guidelines”. Accordingly, the goal is not exactly that described in the name of the package. In other words, I believe that the aim should be more specific: to generate recommendations/guidelines based on living syntheses of evidence, not just to produce such syntheses of evidence, which are important but just an intermediate product.
2. **A couple of typos:**

- **Actions of Phase 1 / 5. Consultation with expert methodologist (page 7 of pdf).** This should be numbered as 4, not as 5.
- **Phase 2: Training in living evidence tools and strategies (last paragraph, page 8 of pdf). **Additional training workshops are complementary, not complimentary.
- **Phase 3. Developing real world living evidence synthesis for KT products (last line, left column, page 8 of pdf).** I think it should read “..operating procedures will be available to..”, or “..operating procedures will be made available to..”, rather than “..operating procedures will make available to..”.

Is the study design appropriate for the research question?

Yes

Is the rationale for, and objectives of, the study clearly described?

Yes

Are sufficient details of the methods provided to allow replication by others?

Partly

Are the datasets clearly presented in a useable and accessible format?

Not applicable

Reviewer Expertise:

Clinical epidemiology; knowledge translation; clinical practice guidelines; systematic reviews

I confirm that I have read this submission and believe that I have an appropriate level of expertise to confirm that it is of an acceptable scientific standard.

# Peer review: Reviewer response for version 1

**DOI:** 10.21956/openreseurope.15132.r28062

**Published:** 2021-12-07

**Authors:** Juan Manual Lozano

**Affiliations:** 1 Department of Medical and Population Health Sciences Research, Herbert Wertheim College of Medicine, Florida International University, Miami, FL, USA

Article: Implementing living evidence to inform health decisions: a strategy for building capacity in health sector (Protocol).

 Authors: María Ximena Rojas-Reyes, Gerard Urrutia Chuchí, Gabriel Rada, Pablo Alonso, David Rigau.

 The article describes the plan of a project aimed at developing and evaluating a strategy for capacity building of professionals from scientific organizations, hospital institutions, and HTA agencies (mostly from Spain) on how to use “Live Evidence” (LE). Through the initiative those professional will receive training on the synthesis and dissemination of knowledge transfer [KT] products (structured evidence summaries, health technology assessment reports, or evidence-based recommendations for clinical practice guidelines).

 The researchers propose three phases for their project: 1) developing an LE implementation framework by reviewing the literature, brainstorming meetings, user testing and expert consultation; 2) training the health professionals on the use of LE tools and strategies (specifically the Epistemonikos Living Overview of the Evidence - L.OVE – platform); and 3) supporting them in developing LE synthesis for KT-products, thus applying the framework to real-life situations.

 The article is well written and describes in detail most procedures proposed by the investigators, particularly those for the first step. I have a couple of concerns / suggestions:

1. During phase 1 of the project the researchers propose developing a framework for the implementation of LE. The methods for this step include a review of the literature to identify previous publications on the methodology of LE, meetings of experts to brainstorm on the challenges and solutions on developing living evidence synthesis, a systematic literature review of living systematic reviews, and consultations and a test of the framework with experts from several external organizations. For phase 2 the researchers propose to train participants from the health services on the use of the Epistemonikos L.OVE platform. It is not clear to me if these first two phases of the project are supposed to be connected, and if so, how. As described, it seems that they are independent because the platform for phase 2 is already available and ready to use, without the need of completing phase 1. I believe that the authors should clarify the relationship (if any) between the framework developed in phase 1 and the platform proposed for phase 2.
2. The protocol describes several strategies for evaluating the training workshops, the usability of the Epistemonikos tools and its L.OVE platform, and the implementation of the LE framework for creating the KT products (structured evidence summaries, health technology assessment reports, or evidence-based recommendations for clinical practice guidelines) that those working at the health system are expected to develop. My concern is that all these evaluations are centered on the processes and are based only on surveys and interviews aimed at those recruited for participating in the study. Perhaps the authors could consider an evaluation of the products developed during the project as well.3.

Finally, two minor comments regarding points that could be clarified in the text:

- In Fig. 1 the authors mention “Screening TiAB Selection”. What does that mean? What does TiAB stand for?
- Evaluation 3 includes assessing the “clarity, completeness and utility of the Manual of Standardized Operating Procedures and its instructions”. What is this manual? Is that the framework for the implementation of LE developed during phase 1 of the project?

Is the study design appropriate for the research question?

Yes

Is the rationale for, and objectives of, the study clearly described?

Yes

Are sufficient details of the methods provided to allow replication by others?

Partly

Are the datasets clearly presented in a useable and accessible format?

Not applicable

Reviewer Expertise:

Clinical epidemiology; knowledge translation; clinical practice guidelines; systematic reviews

I confirm that I have read this submission and believe that I have an appropriate level of expertise to confirm that it is of an acceptable scientific standard, however I have significant reservations, as outlined above.

# Response

**Published:** 2022-05-10

**Authors:** Maria Ximena Rojas Reyes

**Affiliations:** INSTITUT DE RECERCA DE L'HOSPITAL DE LA SANTA CREU I SANT PAU FUNDACION - IR-HSCSP, Spain

Response to the Reviewer #1 (Reviewer comments in italics)  

***Comment 1:*** *During phase 1 of the project, the researchers propose developing a framework for the implementation of LE. The methods for this step include a review of the literature to identify previous publications on the methodology of LE, meetings of experts to brainstorm on the challenges and solutions to developing living evidence synthesis, a systematic literature review of living systematic reviews, and consultations and a test of the framework with experts from several external organizations. For phase 2 the researchers propose to train participants from the health services on the use of the Epistemonikos L.OVE platform. It is not clear to me if these first two phases of the project are supposed to be connected, and if so, how. As described, it seems that they are independent because the platform for phase 2 is already available and ready to use, without the need of completing phase 1. I believe that the authors should clarify the relationship (if any) between the framework developed in phase 1 and the platform proposed for phase 2.*

**Response:** Thanks for this, you are right, phases 1 and 2 are not connected. Recruitment of health sector organizations and the training of their staff in the Epistemonikos tools and L.OVE platform as technological enable for the living process will take place at the same time phase 1 is running. Phase 3 will start once both previous phases were completed. To address your comment we have included a new figure showing the project development structure (See figure 1), and added the following sentence at the beginning of the phase 2 description: **“This phase will run concurrently with phase1, once we have completed the recruitment process of participating organizations and their members”**

***Comment 2:*** *The protocol describes several strategies for evaluating the training workshops, the usability of the Epistemonikos tools and its L.OVE platform, and the implementation of the LE framework for creating the KT products (structured evidence summaries, health technology assessment reports, or evidence-based recommendations for clinical practice guidelines) that those working at the health system are expected to develop. My concern is that all these evaluations are centered on the processes and are based only on surveys and interviews aimed at those recruited for participating in the study. Perhaps the authors could consider an evaluation of the products developed during the project as well. My concern is that all these evaluations are centered on the processes and are based only on surveys and interviews aimed at those recruited for participating in the study. Perhaps the authors could consider an evaluation of the products developed during the project as well.*

**Response:** This is a very good suggestion; never the less we found the following difficulties to perform the proposed evaluation: Firstly, during phase 3, we, the investigator team, will act as the advisor and co-workers in the development of the living evidence synthesis that organizations will carry out. Therefore, the evaluation of these products should be carried out by a third party, not involved in the process. Secondly, due to its recent emergence, the living evidence model is still under development, there is currently no valid tool that allows a fair and reproducible evaluation. This is part of what we want to achieve with the framework, to generate clear guidelines for what has today been defined as "good practice” in the process. Although we were able to carry out the evaluation by a third party, we consider that the second point would limit the validity of this evaluation.  

***Comment*** *3. Finally, two minor comments regarding points that could be clarified in the text:*

- *In Fig. 1 the authors mention “Screening TiAB Selection”. What does that mean? What does TiAB stand for?*

**Response: **TiAB =titles and abstracts. We added the following footnote to the figure with the explanations of abbreviations *Abbreviations: KT product = knowledge translation product; CPG = clinical rpractice guidelines; HTA= Health teachnology assessment; PICO = population, intervention, comparator, outcome; TiAB =titles and abstracts*

- *Evaluation 3 includes assessing the “clarity, completeness and utility of the Manual of Standardized Operating Procedures and its instructions. What is this manual? Is that the framework for the implementation of LE developed during phase 1 of the project?*

**Response:** Thanks, to address your comment we have included a sentence as part of the phase 3 description, now it looks as follows: “Following the principle of “learning by doing” ^31^, we expect members from the participating organizations to generate at least one living evidence synthesis (i.e., one PICO) needed to develop their own KT products following the LE-implementation framework (see Figure 1 and Figure 2). ***T*o support this task, and ensure that all the organizations' teams are receiving the same information and detailed instructions,  a Manual of standardized operating procedures will make available to the participants in an online repository or as a virtual document.”**

# Peer review: Reviewer response for version 1

**DOI:** 10.21956/openreseurope.15132.r28006

**Published:** 2021-12-06

**Authors:** Ingrid Kremer

**Affiliations:** 1 Maastricht University, Maastricht, The Netherlands

The authors report on the protocol of an elaborate project to build capacity among professionals to implement living evidence to ultimately inform health decisions. This paper has scientific merit, as the authors state that no specific guidelines are available for this type of projects, and aims to deal with an important challenge in current policy and clinical decision making.

 After reading the authors’ paper, a number of questions, minor and major remarks came up:

1. A number of textual errors:
  1. Abstract: *health care interventions* > *healthcare interventions*, *KT-products* > *KT products* (throughout the paper), *capacity building strategy* > *capacity-building strategy*, *region for incorporate* > *region to incorporate*.
  2. In abstract “Knowledge transfer products” was used while in the text “knowledge translation products” was used. Use one or the other.
  3. In last sentence of fourth paragraph of introduction: *based in must *> *based on most.*
  4. Last sentence Grade-approach *: now days *> *nowadays*
  5. Epistemonikos project section:
    1. Epitemonikos > Epistemonikos (first paragraph)
    2. Last paragraph: time consuming > time consumption
  6. Fourth paragraph of part 3, phase 1: gave > given.
  7. I am not familiar with the phrase “essential feather”
  8. Part 4 User testing: transcript > transcribed.
  9. Review “the KT products to be worked could be”
  10. Figure 1: HTA reposrt > HTA report
  11. Evaluation 3: “will be invited to respond to the online survey.”
  12. Ethical issues, 3 paragraph: to participate on > to participate in
2. Plain language summary: first sentence of the second paragraph is too complex. Please consider rephrasing.
3. In the abstract, lay summary and in the expected results the authors state that the LE-implementation framework would be applicable to any country. However, there is no discussion of the framework’s potential transferability to other countries. No specific attention seems to be paid to the transferability of the model throughout the developmental process either. Moreover, recruitment of participants will only be done among employees of Spanish institutions. The extent to which the framework is expected to be transferable due to differences in professional training and country-specific standards or structures should be discussed more elaborate.
4. Include reference to substantiate the statement: “There is still a gap among healthcare professionals in producing and using the most current evidence for decision-making.”
5. Include reference to substantiate the statement: “Nowadays, it is not uncommon to find more than one published SR that answers the same question, often reaching different conclusions.”
6. Second specific objective: should “and structured evidence summaries” be “KT products”?
7. Overall, the methods sections provides a general overview of the elaborate study project, but some methodological aspects have not been explained on several occasions, specifically, content of interview (guides) and surveys and the type of analysis (quantitative, qualitative, theoretical framework?) of collected data in the reviews and evaluations.
8. It is unclear to what part(s) of the entire project the “study population” in the methods section apply. Moreover, due to the elaborateness of the project, the structure is a bit lost for me at some points. A figure presenting the overall structure of the project and including all different aspects would support the comprehension.
9. The reporting on the study population should be more specific: Does it only include clinical healthcare professionals or members of HTA organizations and researchers? Further eligibility criteria for these professionals have not been reported either. For recruitment a couple of aspects remain unclear: Who is contacted at the organization? Are all organizations in a setting approached, and if not, how will the organizations be selected? How are the healthcare professionals within the organization recruited?
10. Has a librarian been involved in developing the search strategy for part 1 of phase 1?
11. Google scholar seems an unusual choice for a search engine in a literature review. Please provide an explanation.
12. The connection between part 3 and the classification of the screening in part 1 is not clear. Does part 3 make use of records identified classified as b)?
13. Methods for analysis of the results of the data collected through the reviews in part 1 and 3 of phase 1 were not reported.
14. Does the earlier specified study population apply for the user testing?
15. “We expect the LE-implementation Framework will guide groups to use appropriate criteria for defining which clinical problems (structured into clinical questions) benefit from a constant review of the new evidence, the frequency with which these processes should be carried out, and whether to incorporate the new evidence synthesis and conclusions to the KT-products the evidence support.” These aspects have not been discussed previously in any of the methodologies. To facilitate these expectations, I would suggest for these aspects to have a more prominent focus in the development, i.e. in interview guides, data extraction forms for reviews, etc .
16. The heading “data collection and analysis” does not seem to apply for the first part of the section: “To achieve … regularly produce.” Additionally, consider rephrasing “To achieve the capacity building strategy assessment goal, several measures have to take place during the whole process in order to assess” to something in the line of “Before commencing the development of real-world living evidence synthesis for KT products, the development of the LE implementation framework, the training in Epistemonikos tools and the capacity-building strategy for health system organizations should be completed.
17. For sample size for interviews, data saturation needs to be considered.

Is the study design appropriate for the research question?

Yes

Is the rationale for, and objectives of, the study clearly described?

Yes

Are sufficient details of the methods provided to allow replication by others?

Partly

Are the datasets clearly presented in a useable and accessible format?

Not applicable

Reviewer Expertise:

Specific expertise in health economic evaluation studies and a general expertise in quantitative and qualitative research methodologies and systematic literature reviews.

I confirm that I have read this submission and believe that I have an appropriate level of expertise to confirm that it is of an acceptable scientific standard, however I have significant reservations, as outlined above.

# Response

**Published:** 2022-05-10

**Authors:** Maria Ximena Rojas Reyes

**Affiliations:** INSTITUT DE RECERCA DE L'HOSPITAL DE LA SANTA CREU I SANT PAU FUNDACION - IR-HSCSP, Spain

(Reviewer comments in italics)

***Comment 1:*** *After reading the authors’ paper, a number of questions, minor and major remarks came up:*

*A number of textual errors:*

1.
  1. *Abstract: health care interventions > healthcare interventions, KT-products > KT products (throughout the paper), capacity building strategy > capacity-building strategy, region for incorporate > region to incorporate.*
  2. *In abstract “Knowledge transfer products” was used while in the text “knowledge translation products” was used. Use one or the other.*
  3. *In last sentence of fourth paragraph of introduction: based in must > based on most.*
  4. *Last sentence Grade-approach: now days > nowadays*
  5. *Epistemonikos project section:*
    1. *Epitemonikos > Epistemonikos (first paragraph)*
    2. *Last paragraph: time consuming > time consumption*
  6. *Fourth paragraph of part 3, phase 1: gave > given.*
  7. *I am not familiar with the phrase “essential feather”*
  8. *Part 4 User testing: transcript > transcribed.*
  9. *Review “the KT products to be worked could be”*
  10. *Figure 1: HTA reposrt > HTA report*
  11. *Evaluation 3: “will be invited to respond to the online survey.”*
  12. *Ethical issues, 3 paragraph: to participate on > to participate in*

**Response:** Thanks for this, all your suggestions have been included in the text. 

***Comment 2:*** *Plain language summary: first sentence of the second paragraph is too complex. Please consider rephrasing.*

**Response:**  Thanks. We have rephrased it as follows:

**This project seeks to build capacity among health sector organizations working to support health decision making, in the incorporating of the “Living evidence” (LE) approach to the resolution of relevant and rapidly changing clinical questions. We will identify health technology assessment agencies, guideline-developing organizations and secondary and tertiary health care institutions (hospitals) from Spain and Europe. The target population is professionals, members of the technical teams of these organizations, in charge of developing evidence syntheses products aimed at informing clinical and/or policy decisions** *.*   

***Comment 3:*** *In the abstract, lay summary and in the expected results the authors state that the LE-implementation framework would be applicable to any country. However, there is no discussion of the framework’s potential transferability to other countries. No specific attention seems to be paid to the transferability of the model throughout the developmental process either. Moreover, recruitment of participants will only be done among employees of Spanish institutions. The extent to which the framework is expected to be transferable due to differences in professional training and country-specific standards or structures should be discussed more elaborate.*

**Response:** Thanks to the way the LE-implementation framework will be developed, we consider that it will be applicable by organizations of any country or region, for the following reasons:

- It is a framework that guides a methodological process; the model to be implemented (the LE model) is a theoretical model that has been previously proposed by several authors and therefore, unlike decision tree models or Markov models used in the economic analysis, the concept of "transferability” between jurisdictions or regions (as is the case here) does not apply to this particular model.
- As part of its development, a bibliographic review will be carried out without language or region restrictions.
- For the consultation, a group of international expert advisors working in the field of LE from around the world will be made-up.
- Among the participating organizations that will be enrolled for its evaluation, there will not only be Spanish organizations, but also European organizations. These actions will ensure the international character of the framework.

In response to your comment, we have added the following sentence in the description of phase 1 methods: “ **It is expected that the final framework can be suitable to be used by organizations from any country or region, therefore in its development as well as its evaluation, we will involve international expert advisors, health sector organizations from different countries in Europe, and potential users from around the world”**

 Regarding the extent to which the framework is expected to be transferable due to differences in professional training and country-specific standards, our framework will provide a guide for organizations to implement the Living evidence model in the processes they usually follow to produce evidence synthesis, for which international standards have been developed and are widely recognized. Therefore, it assumes basic training in the evidence synthesis methodology as the minimum requirement, which professionals who work in this area are expected to have. In response to your comment, we have added the following sentence in the description of phase 1 methods: “ **The framework will be designed as a tool for organizations to apply as part of the usual processes they follow to develop evidence synthesis. A set of instructions will be provided for each framework section. Nevertheless, we assume the potential user has basic training in evidence synthesis methodology, as the minimum requirement that professionals who work in this area are expected to have.”**

 As we already mentioned, unlike the economic evaluations models, the concept of "transferability" does not apply to the theoretical model been implementing in this project; nevertheless, the extent to which the final framework is expected to be applied due to differences in professional training and country-specific standards or structures will be discussed more elaborately in the paper reporting its development once we have obtained its evaluation results and the feedback from participant organizations and its members.  

***Comment 4*** *: Include reference to substantiate the statement: “There is still a gap among healthcare professionals in producing and using the most current evidence for decision-making.”*

**Response:** We have added the following references:

- van de Goor I, Hämäläinen RM, Syed A, et al. Determinants of evidence use in public health policy making: Results from a study across six EU countries. Health Policy. 2017;121(3):273-281. doi:10.1016/j.healthpol.2017.01.003
- Loncarevic N, Andersen PT, Leppin A, Bertram M. Policymakers’ Research Capacities, Engagement, and Use of Research in Public Health Policymaking. International Journal of Environmental Research and Public Health. 2021; 18(21):11014. https://doi.org/10.3390/ijerph182111014

***Comment 5:*** *Include reference to substantiate the statement: “Nowadays, it is not uncommon to find more than one published SR that answers the same question, often reaching different conclusions.”*

**Response:** We have added the following reference: Vavken P, Dorotka R. A systematic review of conflicting meta-analyses in orthopaedic surgery. Clin Orthop Relat Res. 2009;467(10):2723-2735. doi:10.1007/s11999-009-0765-2 Factors underpinning discrepant findings from reviews on the same topic: A systematic analysis of Cochrane and non-Cochrane reviews. Claudia Hacke, David Nunan bioRxiv 574046; doi: https://doi.org/10.1101/574046

***Comment 6:*** *Second specific objective: should “and structured evidence summaries” be “KT products”?*

**Response: **Yes, agree we have modified the second objective accordingly.

***Comment 7:*** *Overall, the methods sections provide a general overview of the elaborate study project, but some methodological aspects have not been explained on several occasions, specifically, content of interview (guides) and surveys and the type of analysis (quantitative, qualitative, theoretical framework?) of collected data in the reviews and evaluations.*

**Response:** We do not present the script of the interviews because this is part of the developments that will take place during the conduction of the project. From the training and other processes taking place in phases 2 and 3, it will be possible to further enrich the evaluation process. Nevertheless, the general approach for the analysis of data to be obtained as part of each proposed evaluation is already presented in the protocol, as follows:

 Evaluation 1: Data obtained from these surveys will be analyzed using summary measures (e.g. proportion of participants who answer each of the five Likert scale options). These data will allow researchers to redefine this part of the capacity-building strategy (if necessary). Training workshops can also be re-evaluated accordingly.

 Evaluation 2: Data obtained from this survey will be analyzed using summary measures (e.g. proportion of participants who answer each of the five Likert scale options).

 Evaluation 3: The data obtained from these interviews will be included anonymously in the Nvivo® (version 12) 28 program by the same third party and analyzed as qualitative data by an expert in the subject.

 Evaluation 4: Data obtained from these surveys will be analyzed descriptively using summary measures (e.g. proportion of participants answering each item option in categorical variables and median or mean with interquartile range or standard deviation for continuous variables).

***Comment 8:*** *It is unclear to what part(s) of the entire project the “study population” in the methods section applies. Moreover, due to the elaborateness of the project, the structure is a bit lost for me at some points. A figure presenting the overall structure of the project and including all different aspects would support the comprehension.*

**Response**: The study population described in the methods section will be enrolled for phases 2 and 3. We have included a new figure showing the project development structure (See figure 1). And added the following sentence beginning the phase 2: ***“*This phase will run concurrently with phase1, once the recruitment process of participating organizations and their members has been completed.”**

***Comment 9:*** *The reporting on the study population should be more specific: Does it only include clinical healthcare professionals or members of HTA organizations and researchers? Further eligibility criteria for these professionals have not been reported either. For recruitment a couple of aspects remain unclear: Who is contacted at the organization? Are all organizations in a setting approached, and if not, how will the organizations be selected? How are the healthcare professionals within the organization recruited?*

**Response**: We have rewritten the “Sampling” section, as follows:

**We will seek to involve three types of organizations from the health sector that fulfill the characteristic of synthesizing evidence to develop KT products aimed at supporting health decision-making. 1) Health technology assessment [HTA] agencies; 2) secondary and tertiary healthcare institutions (hospitals) involved in institutional HTA programs and 3) clinical practice guideline [CPG] development organizations. The identification of these organizations will be done through the networking relationships that the IIB Sant Pau has, where the Ibero-American Cochrane Center operates, which is recognized in Europe for its different developments in the area of evidence synthesis, and support for the evidence-based medicine. Its relations of interest for this project include: scientific societies and organizations of the Spanish Biomedical Research Network Consortiums (CIBER) ^21^, clinical guideline development organizations in Europe, and hospitals participating in a program that promotes evidence-based informed policies in healthcare institutions in Spain ^22^ The director or leader in charge of the evidence synthesis team from these organizations will be approached to present the project and invite its organization to take part. We will seek to enroll up to eight organizations, at least two organizations of each type described above, by a convenient sample. Each enrolled organization will be asked to involve at least three members of their organization's technical team in charge of developing evidence synthesis to support decision-making. To take part in the study, the members of organizations should meet the following selection criteria: -           To be a contracted worker in the organization -           To be involved in evidence syntheses development tasks -           To complete and provide the informed consent for individual participation in the study**

***Comment 10:*** *Has a librarian been involved in developing the search strategy for part 1 of phase 1?*

**Response**: Yes, the information specialist from the Iberoamerican Cochrane Center, will be involved in this task. We have added the following sentence: **The information specialist of the Iberoamerican Cochrane Center, will devise the search strategy.**

***Comment 11:*** *Google scholar seems an unusual choice for a search engine in a literature review. Please provide an explanation.*

**Response**: We seek to identify papers that talk about methods and these type of papers do not always appear indexed in MEDLINE. The Role of Google Scholar in Evidence Reviews and its Applicability to Gray Literature Searching has been proposed for this type of articles as a highly sensitive source that can provide additional references.

***Comment 12:*** *The connection between part 3 and the classification of the screening in part 1 is not clear. Does part 3 make use of records identified classified as b)?*

**Response**: For this second version we have withdrawn the “Part 3. Systematic review of the LSRs” from this protocol because the following reasons:

1. Given its aims and scope, we considered it a project in itself, therefore we developed an independent protocol (see Auladell-Rispau A, Bendersky J, Santafe A *et al*. Methodological approaches for developing and reporting living evidence synthesis: a study protocol [version 2; peer review: 2 approved]. Open Res Europe 2022, 1:113 (https://doi.org/10.12688/openreseurope.14044.2).
2. Even though its findings will provide important information about what current authors are doing to carry out living systematic reviews, its results may not be important input for the initial framework development 3) Because of the current proliferation of LSR publications available, it will take too much time to obtain results, so will exceed the timeframe for this project's completion.

 

***Comment 13*** **:** *Methods for analysis of the results of the data collected through the reviews in parts 1 and 3 of phase 1 were not reported*.

**Response**: We have added the following sentence describing the data analysis of part 1: “ **We will perform a descriptive analysis of the data extracted and presented the results in a combination of summary tables and narrative descriptions. Data obtained from these papers will be grouped into categories related to the type of information and the moment in the process in which this information can be applied. This review results will contribute to the development of a preliminary checklist of key methodological aspects that will be used as the starting point for the development of the living evidence implementation framework for health system organizations to use and incorporate the LE methodology.”** As mentioned earlier, part 3 has been withdrawn from this protocol. The methods of this part are now presented in the specific project protocol available at: https://doi.org/10.12688/openreseurope.14044.2.

***Comment 14:*** *Does the earlier specified study population apply for the user testing?*

**Response**: No. The user testing will be running on a different population of potential users. It is already stated in the manuscript, as follows: **“We will invite up to 10 participants from the Cochrane Collaboration, members of evidence synthesis related networks, and potential users to take part in the user testing.”**

***Comment 15.*** *“We expect the LE-implementation Framework will guide groups to use appropriate criteria for defining which clinical problems (structured into clinical questions) benefit from a constant review of the new evidence, the frequency with which these processes should be carried out, and whether to incorporate the new evidence synthesis and conclusions to the KT-products the evidence support.” These aspects have not been discussed previously in any of the methodologies. To facilitate these expectations, I would suggest for these aspects to have a more prominent focus in the development, i.e. in interview guides, data extraction forms for reviews, etc .*

**Response**: There are several authors who have proposed developments and methodological approaches for the LE process and this is what we want to capture with the review of the methodological papers. Therefore we cannot indicate with greater precision the variables to be extracted in the review. Items to be included as part of the framework will arise from the literature review, as well as from the brainstorming and expert consultation meetings. The way of structuring the framework will come from the joint construction with the research team and the experts in the field. To address your comment, we have added the following paragraphs as part of the methods description of phase 1.

- At the beginning of the phase description

**An initial framework (LE-implementation framework) will be developed aimed to guide groups to use appropriate criteria for defining which clinical problems (structured into clinical questions) benefit from a constant review of the new evidence, the frequency with which these processes should be carried out, and whether to incorporate the new evidence synthesis and conclusions in the KT products this evidence supports (i.e. CPG, HTA, and structured evidence summaries for health policies). The framework will be designed as a tool for organizations to apply in the usual processes they follow to develop evidence synthesis**

- As part of 1. Review of methodological articles

**Data obtained from these papers will be grouped into categories related to the type of information and the moment in the evidence synthesis process in which this information can be applied. We will perform a descriptive analysis of the data extracted and presented the results in a combination of summary tables and narrative descriptions. Results of this review will contribute to the development of a preliminary checklist of key methodological aspects that will be used as the starting point for the development of the LE implementation framework for health system organisations to use and incorporate the LE model in the synthesis of evidence to inform health decisions** *.*

- As part of 2. Brainstorming meetings description we added:

**Based on the suggestions obtained during this meeting, we will develop the first draft of the framework. We will structure the framework in sections according to the time in which they must be applied during the evidence synthesis process (i.e. at the time of defining the relevant questions to be answered through a living evidence synthesis; when planning the evidence synthesis; during the evidence surveillance and monitoring process, when integrating new eligible evidence and at the time of publication of updates, etc.). Each section will include a series of guidance questions defining each step of the process. In a second meeting, we will present the draft of the framework in order to collect new ideas and innovative suggestions for its improvement.**

***Comment 16:*** *The heading “data collection and analysis” does not seem to apply for the first part of the section: “To achieve … regularly produce.” Additionally, consider rephrasing “To achieve the capacity building strategy assessment goal, several measures have to take place during the whole process in order to assess” to something in the line of “Before commencing the development of real-world living evidence synthesis for KT products, the development of the LE implementation framework, the training in Epistemonikos tools and the capacity-building strategy for health system organizations should be completed*.

**Response**: We have erased the first part of the section and included your suggestion as follows: **Several measures will take place during the whole project in order to assess: the LE-implementation framework; the training workshops on Epistemonikos tools; and the capacity-building strategy used for participant health system organizations to be able to implement the “living evidence” process in the development of KT products.**

***Comment 17: **For sample size for interviews, data saturation needs to be considered.*

**Response**: Thanks, we will take this into account.
